# HIV/AIDS in the state of Amazonas, Brazilian Amazon: decentralization, third-party support and community action

**DOI:** 10.1590/0037-8682-0048-2026

**Published:** 2026-08-21

**Authors:** Adele Schwartz Benzaken, Zeca Manuel Salimo, Maria Eduarda Leão Farias, Aretha Gomes Omena, Ronaldo Campos Hallal, Renato Chuster Hamed Humar, Beto de Jesus, Izabella Picinin Safe, Marcus Vinícius Guimarães Lacerda

**Affiliations:** 1 AIDS Healthcare Foundation, Los Angeles, United State of America.; 2 Fundação Oswaldo Cruz, Instituto Leônidas & Maria Deane, Manaus, Amazonas , Brazil.; 3 Universidade Lúrio, Nampula, Moçambique.; 4 Fundação de Medicina Tropical Dr. Heitor Vieira Dourado, Manaus, Amazonas, Brazil.; 5 AIDS Healthcare Foundation, São Paulo, SP, Brazil.; 6 Universidade do Estado do Amazonas, Manaus, Amazonas, Brazil.; 7 Fundação de Vigilância em Saúde do Amazonas Dra. Rosemary Costa Pinto, Manaus, Amazonas, Brazil.

**Keywords:** HIV, AIDS, Brazilian Amazon, Antiretroviral therapy, Combination HIV prevention, Decentralization, Retention in care.

## Abstract

Amazonas is Brazil’s largest state and a paradigmatic tropical medicine setting: vast territories connected mainly by rivers, high cultural and linguistic diversity, and multiple co-endemic infections. The state comprises 62 municipalities and roughly 4.3 million inhabitants, with approximately half of its inhabitants residing in Manaus, the capital, where specialized human immunodeficiency virus (HIV ) services have historically been concentrated. However, the HIV/ acquired immunodeficiency syndrome (AIDS) epidemic has shown sustained interiorization beyond the capital and a high AIDS mortality coefficient, reflecting late diagnosis and gaps in retention in care. Over the last decade, expanded testing, rapid initiation of antiretroviral therapy (ART), the introduction of pre-exposure prophylaxis (PrEP) and post-exposure prophylaxis (PEP), HIV self-testing, and decentralized municipal services in partnership with third parties, such as the AIDS Healthcare Foundation (AHF), have coincided with declining AIDS mortality, yet heterogeneity across municipalities remains pronounced, including in predominantly Indigenous areas and border regions. This review synthesizes key programmatic inflection points and highlights the complementary roles of the public sector, third-party organizations, and civil society in implementing combination prevention, improving linkage to care and monitoring loss to follow-up. In this narrative review, we argue that HIV in the Brazilian Amazon should be understood through a syndemic lens encompassing tuberculosis co-infection, mobility and geographic dispersion, and that progress toward 95-95-95-aligned targets will require differentiated service delivery, integrated tuberculosis (TB)/HIV strategies, and supply-chain and workforce resilience tailored to riverine, rural and Indigenous contexts.

## WHY HIV MATTERS IN THE BRAZILIAN AMAZON

In 2025, according to the Joint United Nations Programme on HIV/AIDS (UNAIDS), an estimated 40.9 million people worldwide were living with HIV (PLHIV)^1^. In 2024, Brazil was estimated to have approximately 39.216 PLHIV^2^. Brazil is among the leading low- and medium-income countries (LMICs) progressing towards achieving the 95-95-95 UNAIDS goals in the coming years^3^. In 2024 the country reached the following 89-82-96, i.e., 89% of PLHIV know their diagnosis, 82% of those are on antiretroviral treatment (ART), and 96% of those have an undetectable viral load^4^.

The Brazilian Amazon is characterized by rapid demographic change and extreme geographic dispersion. In the Amazonas state, long travel distances reliant on river transport and seasonal navigation conditions shape access to health services, while concentrated specialized care in Manaus, the capital, creates structural constraints on referral and follow-up for remote municipalities. These constraints affect every step of the human immunodeficiency virus (HIV) care cascade, from timely diagnosis to repeat visits for laboratory monitoring and medication refills. In 2022, the care cascade in the state was as low as 87-82-76^5^.

For tropical medicine audiences, this region also illustrates how HIV outcomes are influenced by co-endemic infections (notably tuberculosis - TB), climate- and river-dependent mobility, and health-system resilience under shocks such as the COVID-19 pandemic. Lessons from Amazonas state are broadly relevant to other tropical settings where HIV programs must operate over long distances and where there is limited laboratory capacity and intersecting epidemics.

The Amazon context exemplifies a syndemic environment in which co-endemic infections, geographic barriers to healthcare access, institutional constraints, and structural inequities interact synergistically, producing health outcomes that are worse than would be expected from the effect of any single factor alone^6^. This framing, rather than treating HIV as a discrete epidemic requiring vertical programmatic responses, underpins the analytical approach of this review.

## HISTORICAL TRAJECTORY AND THE INTERIORIZATION OF THE EPIDEMIC

A review of the period 2001 to 2012 described marked increases in the incidence of AIDS and AIDS-related mortality in Amazonas state, initially concentrated in Manaus but progressively dispersing to municipalities of the interior (interiorization)^7^. Young adults accounted for most diagnoses and, over time, the epidemic progressively shifted toward a predominance among men, with growth among men who have sex with men (MSM) as well as among heterosexual men. Despite national improvements in HIV diagnosis and treatment coverage, Amazonas state sustained high AIDS-related mortality, consistent with delayed diagnosis and incomplete engagement in care^8^.

Clinical and programmatic observations from the same period suggest plausible mechanisms. Baseline CD4 counts at entry to care were frequently low, indicating late presentation to health systems^7^ ^,^ ^9^. Coinfections typical of the regional tropical disease profile were common, with tuberculosis repeatedly identified as a major driver of severe disease and AIDS-related death. ART availability, while historically concentrated in specialized services in Manaus, has been reported to face geographically uneven and logistically challenging coverage for remote and riverine populations^10^ ^-^ ^12^. Low absolute numbers among Indigenous people might be a result of underreporting, driven by geographic and logistical constraints, and should not obscure heightened structural barriers to prevention and care in areas with linguistic diversity, limited transportation, constrained lab infrastructure, and the lack of trained health professionals^13^.

## METHODS

This work is a narrative review developed in accordance with the principles outlined by the Scale for the Assessment of Narrative Review Articles (SANRA), integrating peer-reviewed literature with publicly available programmatic and surveillance data relevant to the HIV/AIDS response in the state of Amazonas, Brazilian Amazon. Peer-reviewed literature was identified through targeted searches in PubMed/MEDLINE, SciELO and LILACS, using combinations of the terms "HIV", "AIDS", "antiretroviral therapy", "pre-exposure prophylaxis", “post-exposure prophylaxis, "decentralization", "tuberculosis", "Amazon", "Amazonas" and "Brazil", with no formal language or date restrictions, although emphasis was placed on publications from 2015 onward to align with the period of structured national surveillance. 

The scope review encompasses epidemiological investigations, programmatic and policy analyses, and community-based interventions relevant to the decentralization of HIV/AIDS care and third-sector engagement in the region. References were selected based on thematic relevance to the regional epidemic and programmatic innovations. This review adopted a syndemic framing, rather than an exhaustive systematic analysis of all HIV/AIDS-related research and interventions conducted in the state.

Epidemiological and programmatic data were obtained from the Fundação de Vigilância em Saúde do Amazonas Dr. Rosemary Costa Pinto (FVS-RCP) dashboards (https://www.fvs.am.gov.br/ver_painel/6), national databases maintained by the Ministry of Health, including the Department of HIV/AIDS, Tuberculosis, Viral Hepatitis and Sexually Transmitted Infections https://www.gov.br/aids), and the UNAIDS global fact sheets. Operational data on pre-exposure prophylaxis (PrEP) and post-exposure prophylaxis (PEP) dispensation, decentralization of HIV services and the number of PLHIV in routine follow-up were obtained from the Manaus Municipal HIV/AIDS Program and from records shared by the AIDS Healthcare Foundation (AHF) and are presented as observed in routine programmatic reports. Figures were constructed by the authors using data from these sources; the underlying period and source are indicated in each figure legend. Given the narrative nature of this review, no formal risk-of-bias assessment or quantitative synthesis was conducted; instead, the work seeks to provide an integrated programmatic and epidemiological reading of the HIV response in Amazonas, oriented toward tropical medicine audiences.

## RECENT EPIDEMIOLOGY (2015-2024): PROGRESS WITH PERSISTENT HETEROGENEITY

The Amazonas state has consistently recorded acquired immunodeficiency syndrome (AIDS) detection and mortality coefficients higher than the Brazilian national average; however, the most recent indicators suggest a gradual improvement^2^. Figure 1 depicts the number of HIV infections and AIDS cases between 2015 and 2024.

**Figure:**
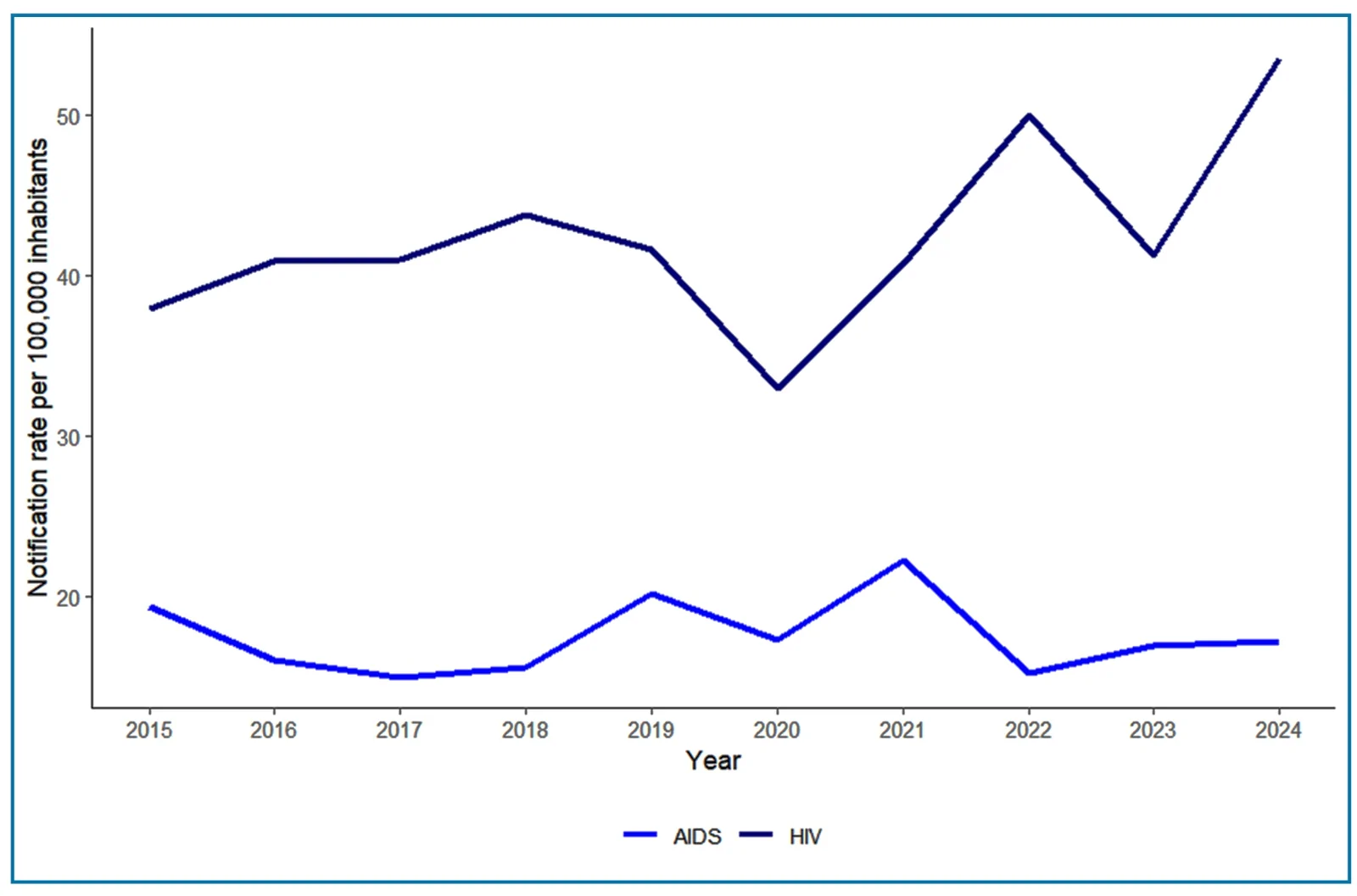

**FIGURE 2:**
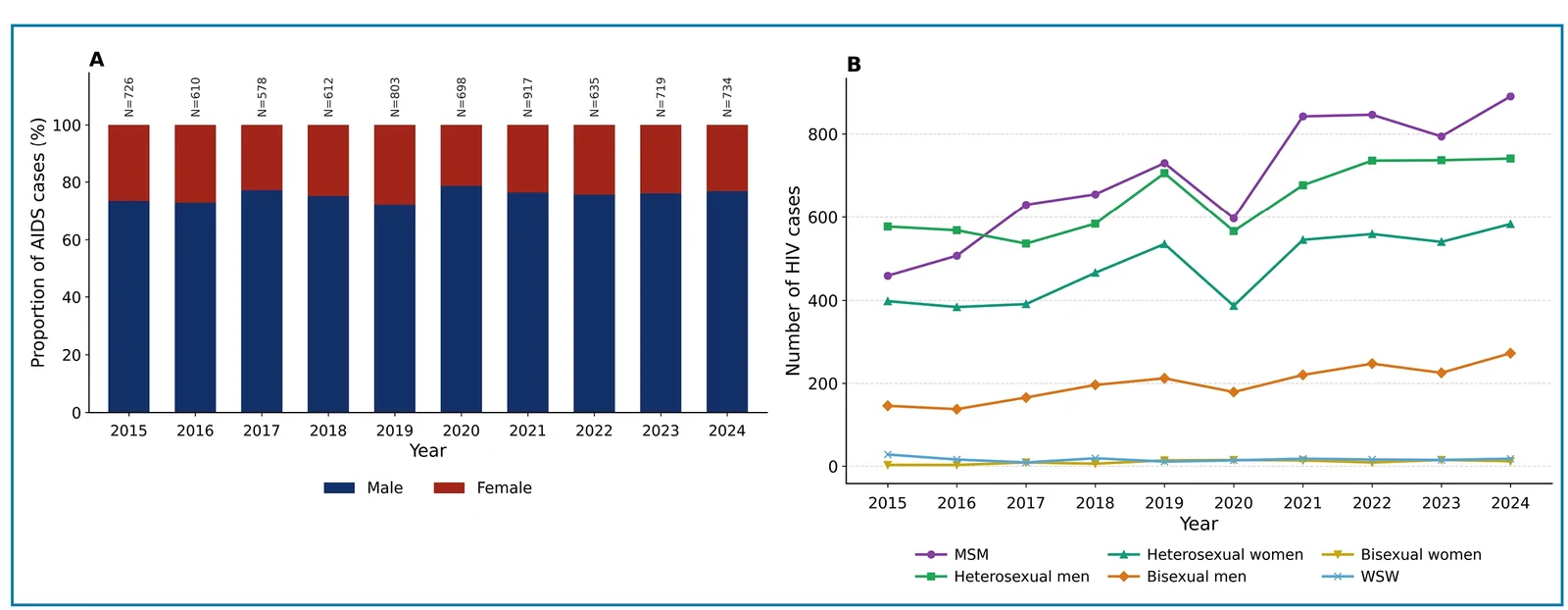
**(A):** Distribution of AIDS cases by sex (2015-2024); **(B):** Number of HIV cases by category of exposure in Amazonas State (2015-2024).

The increase in HIV case detection observed from 2022 onward is more plausibly interpreted as reflecting diagnostic recovery and testing expansion following the COVID-19 pandemic, which caused substantial disruptions to HIV testing services worldwide, also affected the Amazonas and other Brazilian states^14^. The broadening of self-testing distribution and decentralized testing points during this period contributed to increased case ascertainment among previously undiagnosed individuals. Surveillance artefact, including improved notification completeness from newly activated municipal services, may represent an additional contributing factor. Figure 2 shows a stable male predominance, with most cases in MSM. The state has the second-highest incidence of AIDS and the third-highest AIDS mortality rate in the country. However, there was a reduction in AIDS-related mortality in Amazonas between 2013 and 2023, alongside increased HIV case detection in 2023 compared with 2022, a trend interpreted as being due to improved diagnostic coverage and post-pandemic catch-up in testing. 

TB/HIV coinfection remains a critical marker for program performance in the Amazon^12^. Municipalities characterized by difficult geographic access and a high proportion of Indigenous residents, particularly in the northwestern region of the state, possess structural and contextual vulnerabilities that reinforce the need for integrated TB/HIV strategies. This includes bidirectional screening, preventive therapy for latent TB infection, and rapid ART initiation. In addition, intense population mobility within the Amazon basin and across international borders further complicates continuity of care, reinforcing the importance of interoperable information systems and cross-jurisdictional coordination.

Within the syndemic HIV/TB framework, the 2026-2030 agenda should prioritize the consolidation of tuberculosis preventive treatment (TPT) for people living with HIV in remote and Indigenous contexts, addressing two operational dimensions that currently constrain the preventive cascade^15^. The first is stock stability: integrating TPT commodities, including the shorter rifamycin-based regimens (3HP and 1HP) recommended by current WHO guidance^16^, which reduce treatment duration and may improve completion, into existing antiretroviral supply chains, supported by buffer-stock strategies, demand forecasting and decentralized storage adapted to fluvial logistics, in order to mitigate the stockouts that disproportionately affect interior municipalities. The second is adherence support: deploying differentiated, community-based models^17^ delivered in articulation with the Indigenous health subsystem (SasiSUS/SESAI) and its network of Indigenous community health agents, with culturally adapted approaches that prioritize community protagonism rather than externally imposed protocols^13^ ^,^ ^18^. Shorter regimens, combined with community-supported completion and telehealth-enabled monitoring, offer a plausible pathway toward sustainable TPT coverage in these settings, although their effectiveness in the Amazonian context will require prospective evaluation^17^ ^,^ ^19^.

## STRUCTURAL AND OPERATIONAL CHALLENGES IN A RIVER-BASED HEALTH SYSTEM

Four recurrent obstacles emerge in surveillance and service reports: late diagnosis, loss to follow-up, centralized care, and geographic barriers. Long travel times and concentration of specialized services can delay confirmatory testing, baseline evaluation and treatment initiation, and can compromise retention in care. 

Limited access to same-day laboratory support in remote areas contributes to delayed staging and missed opportunities for rapid ART initiation. Some municipalities in the interior have established local networks that can perform rapid viral load testing and CD4 counts. Smaller municipalities, however, remain dependent on sending samples to nearby municipalities or to Manaus. Sample transportation is not always timely, and sometimes there are logistical delays due to geographical or climatic constraints. Stigma, discrimination and violence affecting key populations may further reduce service utilization.

The COVID-19 crisis in Manaus exposed the fragility of supply chains and routine service delivery in the region, with downstream effects on HIV testing and care continuity^20^. These experiences underscore the importance of resilient distribution systems for ART and diagnostics, as well as contingency planning for outbreaks and extreme climate events, and the implementation of differentiated service delivery models (e.g., multi-month dispensing and community-based refill strategies) that are adapted to remote settings.

A further dimension of institutional vulnerability concerns the reliance on non-governmental organizations and project-based support to sustain HIV services. Civil-society organizations, including the AHF and other partners, have contributed substantially to testing, prevention, outreach to key populations, and linkage and adherence support in Amazonas, frequently reaching populations that the formal public network struggles to serve. These contributions are valuable and, in several respects, indispensable to current coverage. 

This concern is particularly salient in Amazonas, where the expansion of third-party engagement has coincided with stalled public hiring, and where Non-Governmental Organization (NGO) substitution has emerged within an otherwise robust national HIV program. Framed through an implementation science lens, the relevant objective is not to diminish the role of civil society, whose proximity, flexibility and reach are genuine assets, but to ensure that third-party support is progressively integrated into, and ultimately backstopped by, institutionalized public capacity.

Poverty and socioeconomic precarity disproportionately concentrated in peripheral urban neighbourhoods of Manaus and in rural and riverine communities of the interior constrain individuals' ability to prioritize health-seeking behaviour, sustain adherence and navigate bureaucratic care systems^21^. 

Structural racism shapes these vulnerabilities unevenly: Indigenous peoples in Amazonas face compounded barriers rooted in historical dispossession, linguistic exclusion from health information, and the chronic underfunding of the Indigenous health subsystem (SasiSUS/SESAI), whose capacity to integrate HIV prevention and care into primary health services remains limited.

Gender inequities, including intimate partner violence, economic dependence and unequal power in sexual negotiation, constrain access to PrEP and PEP and increase vulnerability among cisgender women and transgender individuals. Migration linked to informal extractive economies (fishing, mining, agriculture) and across international borders with Colombia, Peru and Venezuela introduces discontinuities in care and diagnostic coverage that interoperable information systems alone cannot resolve.

Finally, structural stigma, the embedding of HIV-related discrimination within institutional and community norms, continues to reduce service utilization by key populations and delay care presentation across population groups^22^. Operationalizing the syndemic framework in Amazonas therefore requires responses that engage these structural conditions directly, rather than treating them as background context for biomedical interventions.

**FIGURE 3:**
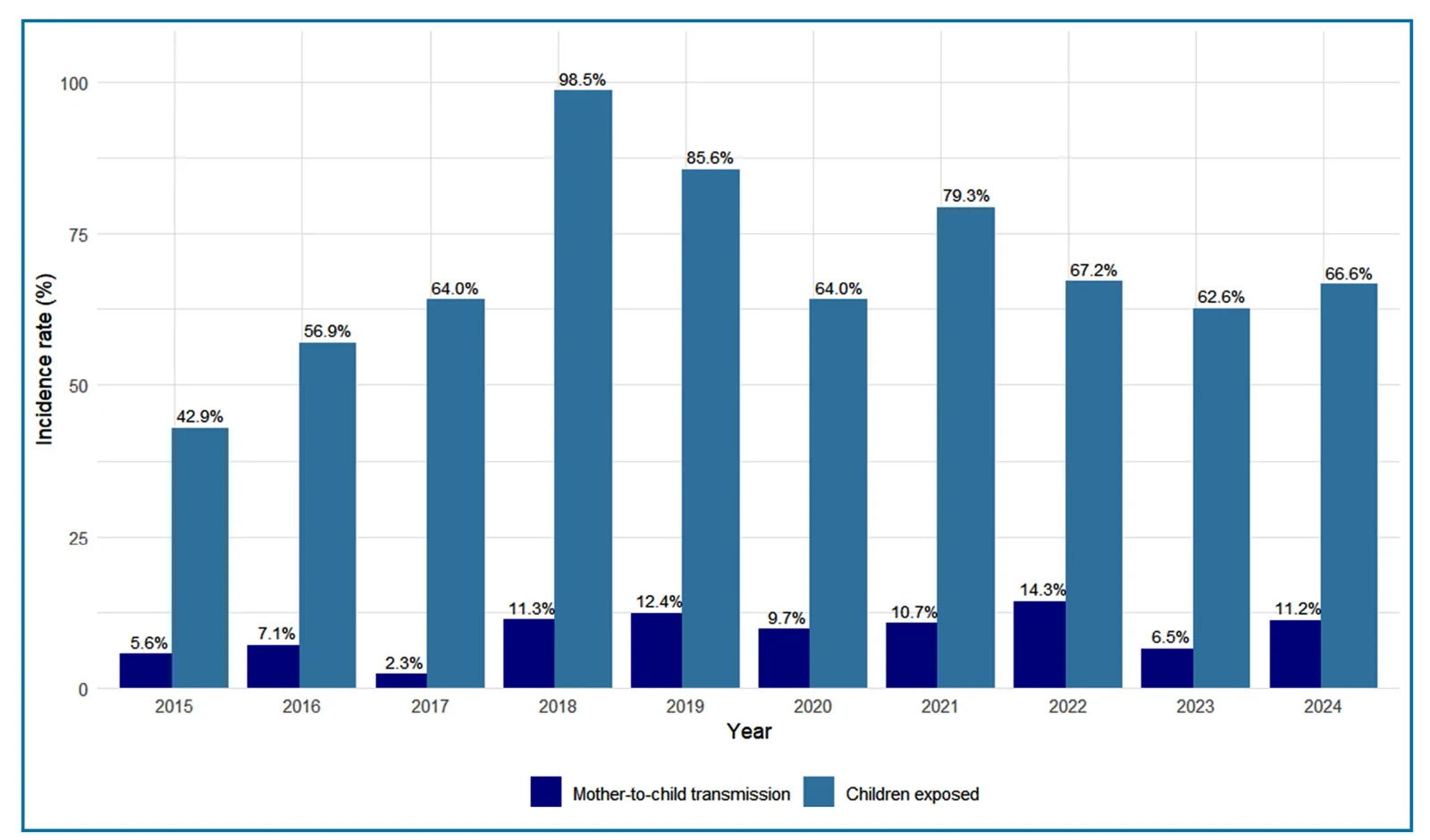
Mother-to-child transmission and Children Exposed in Amazonas State (2015-2024).

Figure 3 shows the number of exposed children and confirmed cases of mother-to-child transmission (MTCT) in Amazonas state. The MTCT rate remains persistently high in Amazonas and the contributing factors include late initiation of antenatal care, loss to follow-up between antenatal and postpartum periods, inconsistent viral load monitoring during pregnancy, and low uptake of PrEP and PEP during breastfeeding, a phase that remains underserved by current prevention protocols. Structural determinants, including poverty, limited transportation, geographic distance from maternity services and inadequate integration between obstetric and infectious disease care are some of the many perceived barriers to access HIV care^23^. Targeted quality improvement and implementation research at the facility and community level are needed to translate existing policy into sustained reduction in transmission rates. In December 2025, Brazil was certified by the WHO as having eliminated as a public health problem and therefore, the surveillance of these cases is critical. Postpartum women are poorly tested for HIV infection, and the frequency of PEP and PrEP in this population is still low, which may lead to infection of children during lactation^24^ ^,^ ^25^. 

## PROGRAMMATIC INNOVATIONS AND SCALE-UP IN PREVENTION AND CARE

Brazil adopted universal ART policies in the early 2010s and subsequently strengthened recommendations for rapid treatment initiation, enabling treatment as prevention and improving population-level viral suppression. The introduction of more effective first-line ART regimens, such as the dolutegravir-based combination, also reduced disease progression and AIDS-related mortality^26^. In Amazonas, the last decade has brought tangible innovation, including expanded HIV testing strategies, a strong drive for decentralization of care in Manaus, enabling rapid ART initiation workflows, dissemination of the Undetectable equals Untransmittable (U=U) message, and the introduction of PrEP in 2018. PrEP implementation was initially anchored at the *Fundação de Medicina Tropical Dr. Heitor Vieira Dourado* (FMT-HVD) in Manaus^27^ and subsequently expanded through additional services. Currently, 26 public health units are dispensing PrEP in Manaus, including in six prisons. Only 1% of the PrEP users are being seen at a private health unit. Outside the capital, 9 of the 61 municipalities (Coari, Tefé, Tabatinga, Humaitá, Lábrea, Maúes, Manacapuru, Parintins and Presidente Figueiredo) are already prescribing PrEP. In total, more than 2,247 people in the whole state are getting PrEP regularly^28^. Qualitative studies conducted in Manaus reinforce the need to clearly differentiate actions related to the treatment of PLHIV from those targeting PrEP users, as failure to do so may lead to discrimination^29^.

Within the public health system, HIV self-testing has relevance in Amazonas, since concerns related to privacy, geographic distance and limited access to health facilities may restrict the uptake of conventional testing services. From January to November 2025, a total of 42,794 self-tests were distributed statewide of which 11,185 were distributed in Manaus^30^.

**FIGURE 4:**
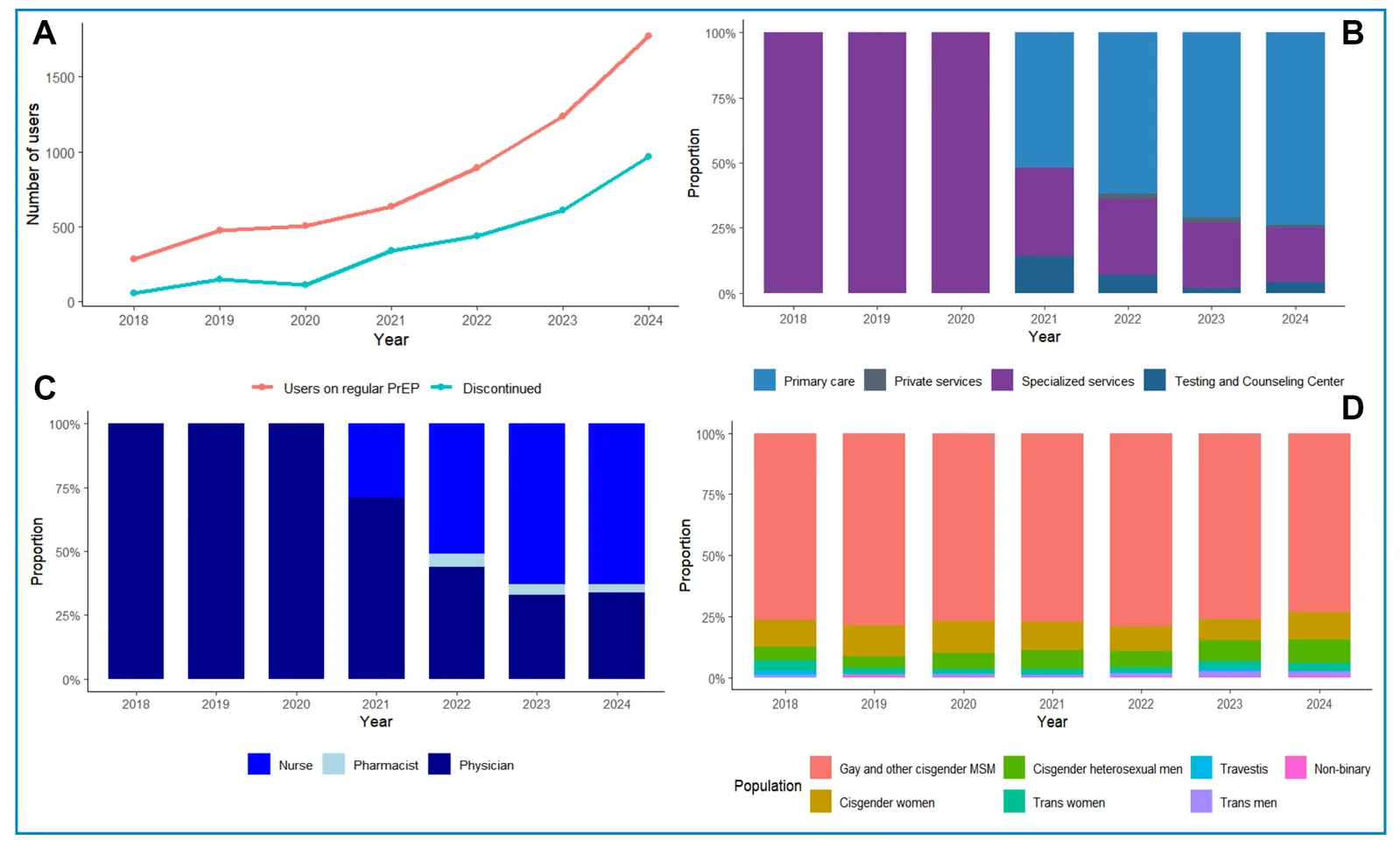
**(A):** Use of Pre-exposure Prophylaxis in Amazonas State cases (2018-2024); **(B):** PrEP Dispensation Services in Amazonas State (2018-2024); **(C):** Prescriptors of PrEP in Amazonas State (2018-2024); **(D):** PrEP users by Population in Amazonas State (2018-2024).

Decentralization has emerged as a critical enabling strategy in the HIV prevention and care continuum. Bringing HIV testing, PrEP/PEP provision and routine ART follow-up into neighborhoods and primary care units can reduce the burden of travel and improve retention in care. Task-sharing approaches, including nurse-led protocol-based PrEP initiation and same-day dispensing workflows, are particularly valuable in settings with limited availability of physicians^31^. Figure 4 illustrates the increased access to PrEP in the state (Figure 4A), highlighting the increase in dispensation in primary care units over time (Figure 4B), the increasing contribution of nurses and pharmacists to PrEP prescription (Figure 4C), and the profile of users (Figure 4D). To maximize its impact, decentralization should be paired with minimum laboratory capability or robust sample-transport systems, as well as clearly defined referral pathways for complex clinical conditions.

**FIGURE 5:**
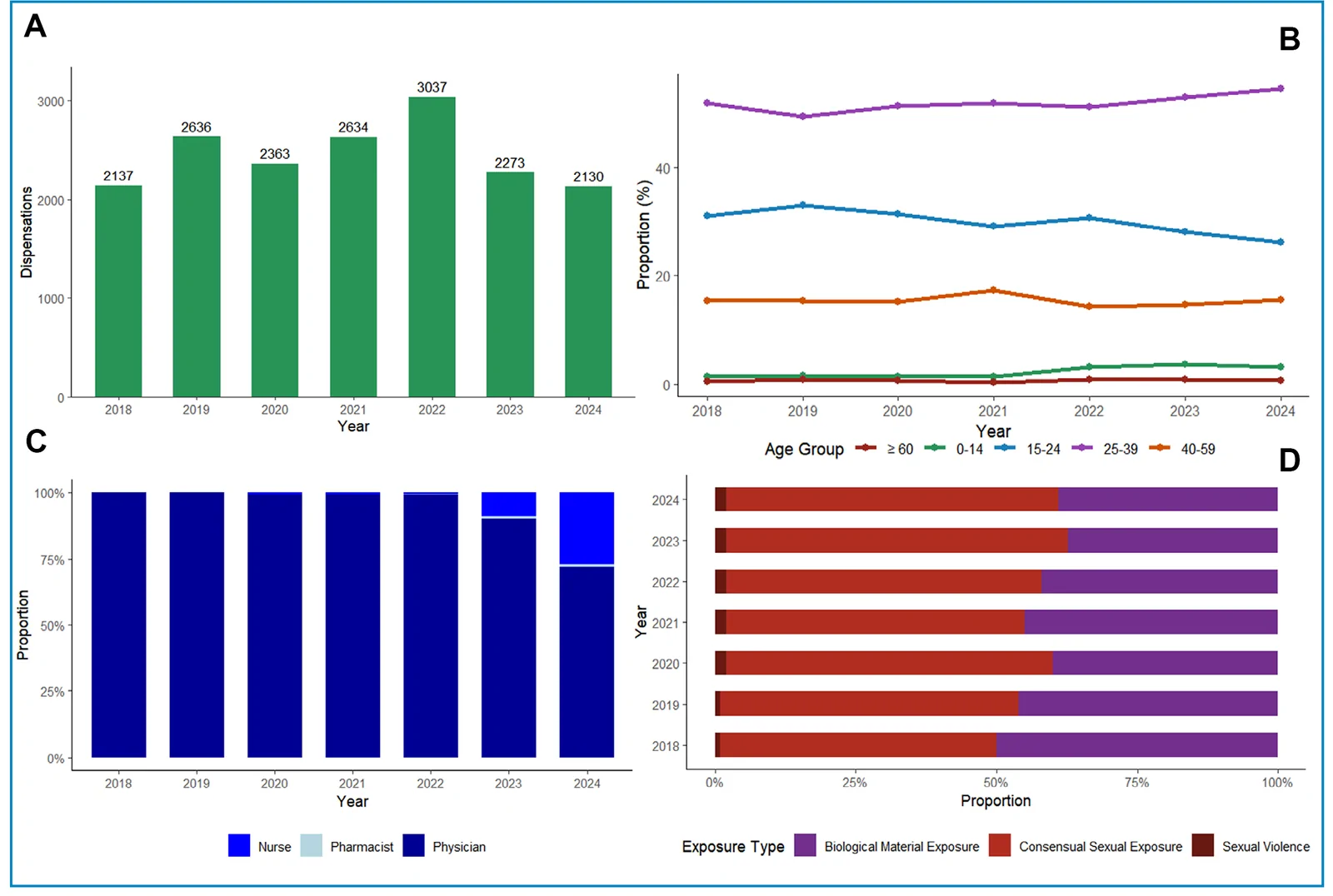
**(A):** PEP Dispensation in Amazonas State (2018-2024); **(B):** Dispensation of PEP by age group in Amazonas State (2018-2024); **(C):** Prescriptors of PEP in Amazonas State (2018-2024); **(D):** Types of Exposure for PEP in Amazonas State (2018-2024).

Figure 5 presents data on HIV PEP in the state of Amazonas, including the number of dispensations over time (Figure 5A ), the distribution of users by age group (Figure 5B), the professional categories responsible for PEP dispensation (Figure 5C), and the types of reported exposure (Figure 5D). The number of dispensations increased from 2018 to 2022 and then dropped until 2024 to a similar level from the start of PEP services. It is shown that the age group stratification has not changed drastically since 2018. The profile of exposure type started equal, but it tends to be more predominantly consensual sexual exposure, which suggests the information regarding PEP services has spread effectively. The observed decline in PEP dispensation after 2022 warrants analytical attention. Plausible explanations include service reorganization within decentralizing systems, gaps in notification from newly activated dispensing points not yet fully integrated into state surveillance platforms, and possible changes in care-seeking behavior following pandemic-period disruptions. It is also possible that some individuals with PEP indications are accessing diagnosis through PrEP services and have their HIV Point-Of-Care Testing along with the PrEP prescription. The underlying driver remains uncertain and should be investigated through prospective monitoring of PEP access, barriers, and reporting completeness.

The STRETCH pragmatic cluster randomised trial in South Africa (Stretch trial) and the CIPRA-SA non-inferiority trial^32^ demonstrated that nurse-initiated and managed antiretroviral treatment (NIMART) achieves clinical outcomes comparable to physician-led care, a finding reinforced by Cochrane synthesis^33^ and progressively incorporated into WHO guidance^34^. Building on this evidence, the Differentiated Service Delivery (DSD) framework has reframed HIV care as a portfolio of contextually adapted models tailored to client characteristics and health system capacities^35^.

In Brazil, ART initiation was historically restricted to physicians under the national Clinical Protocol and Therapeutic Guidelines. To overcome geographic and workforce barriers, the State of Amazonas issued Resolution CIB/AM No. 071/2024, the subnational regulation authorizing trained nurses to prescribe the initial ART regimen for newly diagnosed individuals. This policy aligns with WHO recommendations for rapid treatment initiation^34^ and builds on previous expansions of nursing competencies established by COFEN Opinion No. 259/2016 and COFEN/CTAS Opinion No. 12/2020. Since November 2024, nurses within the Unified Health System (SUS) have been trained to prescribe first-line ART for asymptomatic patients aged 13 or older. Since its implementation, approximately 361 nurse-led prescriptions have been issued, operationalizing the 'test and treat' strategy and promoting immediate or rapid ART initiation within seven days of diagnosis. In 2024, 33% of patients started ART within 7 days. This figure rose to 39% in 2025 and stood at 45% until May 31, 2026^36^.

However, these data strictly reflect implementation indicators (penetration and adoption) rather than clinical effectiveness. Because comparative data on viral suppression and retention in care remain a critical evidentiary gap, a rigorous prospective evaluation is a mandate and a priority. Structuring this assessment as a hybrid effectiveness-implementation design^37^ would allow for the simultaneous capture of clinical endpoints (viral suppression, retention, time-to-initiation, and mortality) and implementation outcomes (acceptability, fidelity, and sustainability)^38^, establishing the necessary evidence base to inform future expansion within and beyond the region.

Severity stratification circuits, known as the Advanced AIDS Circuit in Brazil, are a key strategy for the early recognition of patients with advanced HIV who require immediate initiation of ART, laboratory screening for common opportunistic infections, such as TB, cryptococcosis and histoplasmosis, and timely initiation of prophylaxis. In parts of Brazil, disseminated histoplasmosis remains a frequently underdiagnosed and neglected condition among patients with AIDS, underscoring the need for improved and more accessible diagnostic tools^39^. This strategy was implemented following nearly a decade of a care model that strengthens linkage to care, integrating part of the response to advanced HIV and the social determinants of the epidemic^40^ ^,^ ^41^.

In 2024, for the first time in many years, municipalities in the interior of Amazonas began to provide access to an advanced AIDS circuit^42^. The health professionals in selected municipalities received training to perform the lateral flow lipoarabinomannan assay and cryptococcal antigen test (CrAg), with established referral pathways for lumbar puncture to rule out cryptococcal meningitis in inpatients with positive serum CrAg. In 2025, the municipalities of Tefé and Coari initiated local lumbar puncture procedures and early treatment of cryptococcal meningitis before patient transfer, contributing to improved survival outcomes.

The decentralization of the advanced HIV disease diagnostic package to municipalities such as Tefé and Coari represents a meaningful step toward earlier detection of opportunistic conditions in settings historically dependent on referral to the state capital^42^. As point-of-care assays requiring limited infrastructure, these tests are, in principle, well suited to decentralized care. Their long-term feasibility in riverine territories, nonetheless, warrants a realistic appraisal: sustained deployment depends on uninterrupted reagent supply across extended fluvial distances, storage under controlled conditions during transport, maintenance and quality assurance of associated equipment, and the continuous availability of trained personnel. These requirements intensify when coverage is extended from regional hubs to the smaller, more remote communities within their catchment areas, reinforcing the centrality of supply-chain resilience to the durability of the decentralized model.

## THIRD-PARTY ORGANIZATIONS AND PUBLIC-PRIVATE COLLABORATION

The public health system in Brazil counts on a tripartite funding: the Ministry of Health, which pays for drugs and consumables, and development of guidelines; the government of the state, in charge of the logistical distribution to the municipalities; and the municipality, in charge of the full execution of all actions in the field.

**Supplementary Figure 1:**
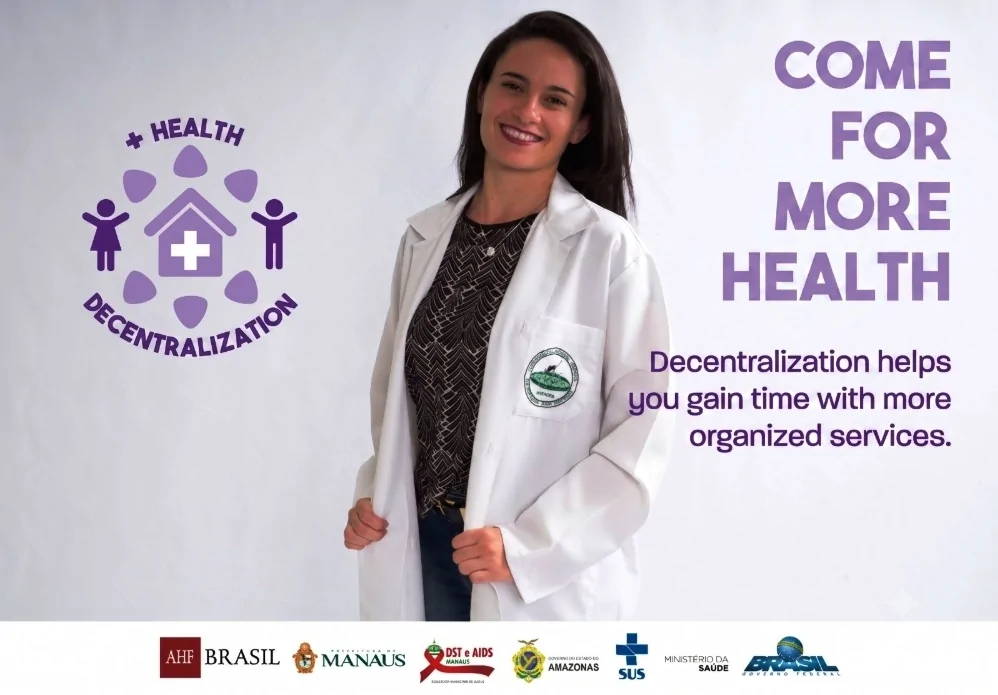
Figure 1

 Third-party organizations, including the AHF, have supported service delivery in Manaus through collaboration with public municipal services since early 2015. One operational model is the deployment of health professionals by the AHF within four municipal care units, and two state care units (FMT-HVD and *Fundação Hospitalar Alfredo da Matta*), including linkage and re-engagement professionals (known as ‘Navigators’). Between 2016 and 2025, the number of PLHIV receiving routine follow-up at municipal services in Manaus increased from 1,339 to 10,589. This expansion coincided with the operational deployment of AHF-supported professionals within municipal care units, a collaboration that is plausibly associated with this growth, while plausible and consistent with implementation goals, cannot be causally isolated from concurrent programmatic developments. The strategy of decentralization relied on a successful population-level campaign coordinated by the AHF, as seen in the advertising folder (Supplementary Figures 1 and Figure 2).

The model aligns decentralization goals with routine monitoring of key indicators such as: (i) the proportion of newly diagnosed patients initiating ART within 14 days; (ii) number of visits and service capacity; and (iii) trends in loss to follow-up. Regularly reviewing indicators as part of routine management can help identify facility-level challenges (staffing gaps, stock-outs, patient tracing needs) and prioritize corrective actions. To ensure sustainability and equity, the long-term value of these partnerships should be assessed transparently through implementation and outcomes research, including cost analyses and patient experience. Patient satisfaction and perception of quality are associated with better ART adherence in Manaus^43^.

While programmatic records document this collaboration's operational successes, the long-term sustainability of embedding externally funded professionals within public health structures requires critical evaluation. Implementation science emphasizes that true institutionalization requires an innovation to be integrated into organizational routines, financing, and governance, independent of external support^38^.

The Manaus model has not yet been formally assessed against these criteria, leaving critical gaps regarding whether quality-improvement processes will persist without NGO presence, and how governance, equity, and patient outcomes are accountable. Addressing these questions through rigorous implementation and cost-effectiveness research is a prerequisite for replicating this model across the Amazonas interior. 

## SUSTAINING DECENTRALIZED HIV CARE: HUMAN RESOURCES AND STRUCTURAL CHALLENGES

Chronic shortages and maldistribution of specialized health professionals constitute a defining structural constraint on HIV care in Amazonas^44^. The State has not conducted a public civil service examination for its health workforce since 2014, with consequent and progressive reliance on temporary and outsourced contracts to sustain service delivery^45^. At reference centers such as the FMT-HVD, this prolonged freeze has compounded attrition through retirement and death. The resulting erosion of clinical and institutional expertise represents a material risk to the stability of the decentralized model. Indeed, task-sharing arrangements such as nurse-initiated ART (Resolution CIB/AM No. 071/2024) and the engagement of third-party actors may be understood, in part, as adaptive responses to this workforce contraction rather than as independent design choices. Recognizing this dependency is essential, as it implies that the sustainability of decentralized HIV care in Amazonas is inseparable from broader workforce-planning decisions, including the resumption of public hiring and the institutionalization of specialized posts at reference centers, which should feature explicitly in the proposed 2026-2030 agenda.

The durability of task-sharing is contingent on continuing education, clinical supervision, and governance arrangements that extend well beyond initial implementation. In the Amazonian context, telehealth-supported supervision and tele-education offer a plausible mechanism to bridge the geographic distance between specialist centers and decentralized prescribers, although their scalability is conditioned by connectivity infrastructure that remains unevenly available across the territory.

According to the Brazilian Medical Demography 2025 report, Amazonas has 6,749 physicians for a population of 4.28 million (1.58 per 1,000 inhabitants), with extreme geographic concentration in the capital. Only 391 physicians serve the interior of the state, corresponding to 0.20 per 1,000 inhabitants, while only 87 infectious disease physicians are available statewide. This distribution reflects profound structural inequities in access to specialist care^44^.

Climate vulnerability adds a further structural dimension. Seasonal flooding, river-level fluctuations and extreme weather events periodically isolate municipalities from supply chains and referral networks, with documented effects on ART continuity and laboratory sample transport^7^ ^,^ ^46^. Digital exclusion constitutes a related barrier: connectivity infrastructure remains insufficient in many interior municipalities, constraining the reach of telehealth-based supervision and mHealth-supported adherence tools to areas where they are most needed. These structural realities must be integrated into logistics planning, resilience strategies and implementation research designs.

Financial and programmatic sustainability also requires attention. Decentralization transfers operational and financial responsibilities to municipalities with limited fiscal capacity, while several community-based activities have depended on short-term externally funded non-governmental actors. Implementation of science literature highlights that sustainability must be planned as an explicit outcome, since withdrawal of external support may compromise coverage and retention gains^38^ ^,^ ^47^. These findings reinforce the need for institutionalized financing, resilient supply chains and structured transition plans toward publicly sustained service delivery.

Accordingly, we frame the municipalization of HIV care not as a completed achievement but as an ongoing process whose long-term success depends on coordinated and sustained investment in workforce, supervision, financing and governance, dimensions that should anchor the proposed 2026-2030 policy and research agenda.

## THE ROLE OF CIVIL SOCIETY: PEER NAVIGATION AND COMBINATION PREVENTION

From 2016 to 2025, civil society initiatives in Amazonas complemented the public health system by operationalizing the community component of combination prevention. Core activities included peer-led education, outreach and risk communication, and linkage support for populations disproportionately affected by HIV and/or facing structural barriers to access. Community-based testing strategies, outreach events and mobile actions helped generate demand for HIV diagnosis, while structured referral pathways promoted timely linkage to clinical services following reactive rapid tests.

Civil society actors, also supported by the AHF in outreach testing activities, contributed to large-scale awareness and prevention campaigns (notably those aligned with the ‘Red December’, and World AIDS Day), integrating counseling, distribution of prevention commodities (including condoms and educational materials), and demand creation for biomedical prevention (PrEP and PEP), where available. In parallel, they engaged in stigma reduction and rights-based advocacy, including participation in public forums and social accountability mechanisms to promote equitable access to prevention, diagnosis and sustained treatment. During the COVID-19 pandemic, community-based psychosocial support and social protection measures (e.g., hygiene supplies and food support) likely helped mitigate vulnerability and supported continuity of care.

## PRIORITY AGENDA FOR RESEARCH AND POLICY (2026-2030)

- Consolidate and evaluate decentralization of HIV prevention and care in Manaus, where most diagnoses still occur, using standardized cascade indicators and equity metrics.
- Empower interior municipalities to deliver HIV care with differentiated service delivery models, multi-month dispensing, and robust referral pathways for complex cases.
- Expand PrEP access across the state (more dispensing units and trained professionals), while monitoring persistence, adherence and equity in uptake among key populations.
- Prepare for long-acting biomedical prevention as evidence and regulatory approvals evolve (e.g., long-acting cabotegravir/lenacapavir), with careful attention to feasibility in remote settings.
- Standardize rapid diagnosis and severity stratification circuits (including point-of-care CD4, where indicated, and streamlined baseline testing) to reduce early mortality.
- Scale up latent tuberculosis infection (LTBI) treatment, empowering nurses and pharmacists to manage and prescribe TPT among people living with HIV, and strengthen bidirectional TB/HIV screening, especially in high-burden municipalities.
- Strengthen logistics for the continuous supply of antiretrovirals, PrEP/PEP, diagnostics and consumables to remote areas, including climate-aware contingency plans.
- Implementation of telehealth services to address geographical and structural access barriers.
- Invest in qualitative and implementation research centered on vulnerable populations (riverine communities, Indigenous peoples, transgender people) to tailor communication, service design and retention strategies.
- Expand and sustain the participation of civil society in prevention, linkage, adherence support and stigma reduction as an essential component of combination prevention.
- Refine the conceptual framework and strengthen causal attribution for disengagement from HIV care, operationalizing the WHO’s four determinant domains (structural, health-system, interpersonal and individual-level factors).
- Strengthen social support interventions, including food security support and differentiated ART delivery (e.g., home delivery of antiretrovirals), when these are identified as drivers of disengagement from care.
- Incorporate explicit transition and integration planning, clearly defined financing responsibilities, and governance arrangements that preserve the complementary strengths of non-governmental actors while reducing the structural dependence of essential services on external project cycles.
- Address the equity gap in multidisciplinary care for PLHIV, including physical rehabilitation and psychosocial support, by exploring task-shared and telehealth-enabled models adapted to the logistical realities of riverine and remote municipalities.

## FINAL REMARKS

Amazonas illustrates both the promise and the limits of Brazil’s HIV response when delivered in a vast tropical region with river-based connectivity, endemic coinfections and deep inequities. The observed improvements in mortality and expanded prevention options represent real progress. Yet late diagnosis, TB coinfection and avoidable loss to follow-up continue to be persistent drivers of morbidity and mortality. Future gains will depend on sustaining decentralization, integrating HIV with TB and primary care, protecting supply chains and supporting community-led strategies that reduce stigma and strengthen engagement. For tropical medicine, the Amazon highlights that HIV control depends as much on geography-sensitive health systems and logistics as on pharmacology.

## Figures and Tables

**Supplementary Figure 2:**
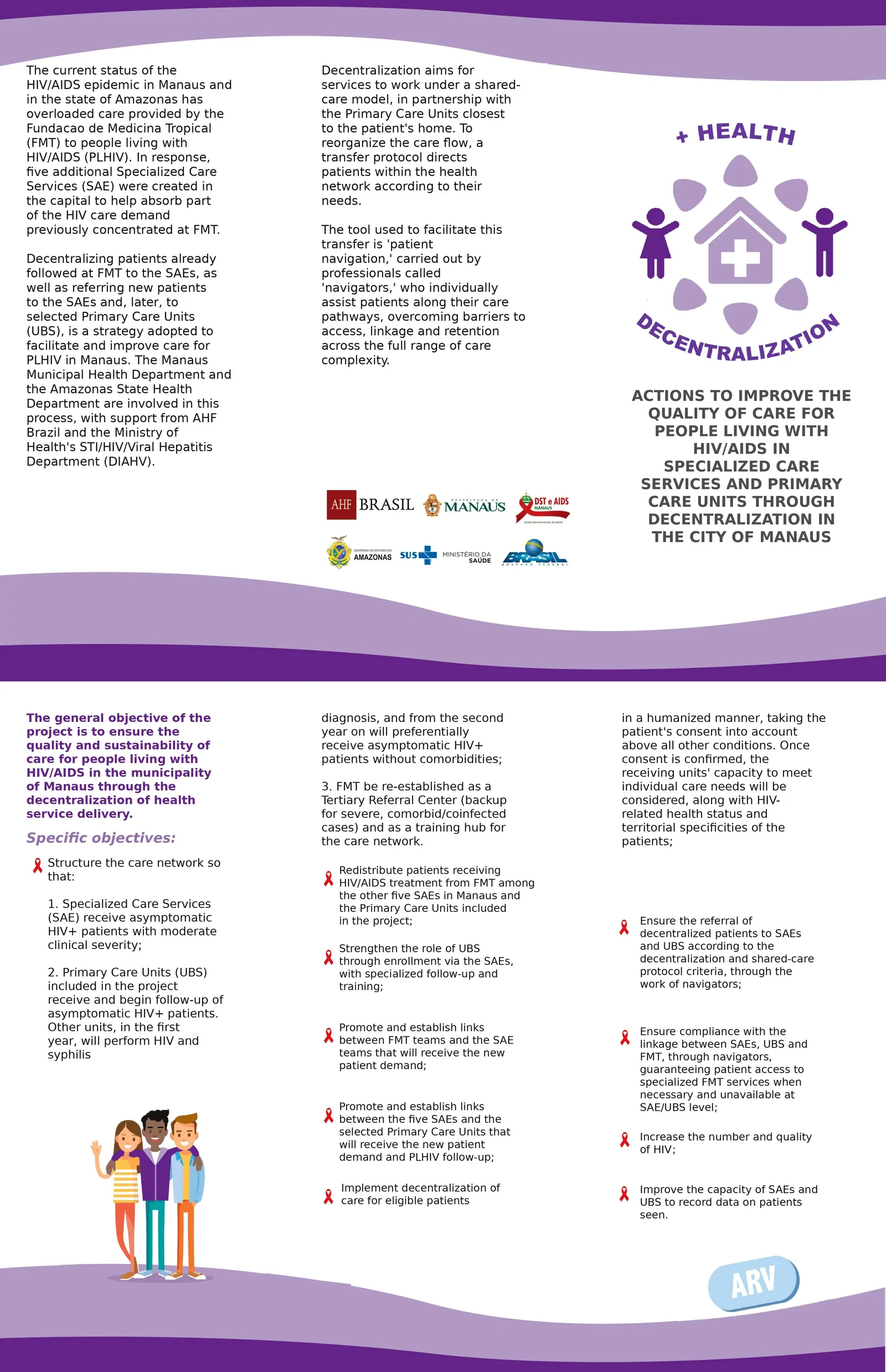
Figure 2

## Data Availability

Data-in-article.
